# Development of Biodegradable Polymer Blends Based on Chitosan and Polylactide and Study of Their Properties

**DOI:** 10.3390/ma14174900

**Published:** 2021-08-28

**Authors:** Ivan Lednev, Evgeniia Salomatina, Svetlana Ilyina, Sergey Zaitsev, Roman Kovylin, Larisa Smirnova

**Affiliations:** 1 Chemistry Department, National Research Lobachevsky State University of Nizhny Novgorod, 23 Gagarin Ave, 603022 Nizhny Novgorod, Russia; ildnv235@gmail.com (I.L.); salomatina_ev@mail.ru (E.S.); ox-eye_daisy23@mail.ru (S.I.); szay@inbox.ru (S.Z.); 2 G. A. Razuvaev Institute of Organometallic Chemistry, Russian Academy of Sciences, 49 Tropinina St., 603950 Nizhny Novgorod, Russia; mulnir@yandex.ru

**Keywords:** mixed composition, chitosan, polylactide, titanium isopropoxide, biocompatibility, mechanical properties, porosity

## Abstract

Composite materials of various compositions based on chitosan and polylactide were obtained in the form of films or porous bulk samples. Preliminarily, poly-d,l-lactide was synthesized by ring-opening polymerization of lactide in the presence of Ti(O^i^Pr)_4_. Polylactide obtained at components molar ratio [lactide]:[Ti(O^i^Pr)_4_] = 3:1 had the best molecular weight characteristics at a high product yield. Film composition with the weight ratio chitosan-polylactide 50:50 wt. % was characterized by high mechanical properties. The value of the tensile strength of the film was 72 MPa with a deformation of 10% and an elastic modulus of 40 GPa, which is higher than the tensile strength of native chitosan by ~three times. The observed effect is a consequence of the fact that the chitosan-polylactide composite has an amorphous structure in contrast to the native chitosan, which is proved by X-ray phase analysis. An increase in the elastic modulus of the composite in the range of 20–60 °C in contrast to polylactide was found by dynamic mechanical analysis. The observed effect is apparently caused by the formation of hydrogen bonds between functional groups of chitosan and polylactide which is possible through an increase in polylactide segments mobility when its glass transition temperature is reached. The composite material is biocompatible and characterized by high cellular adhesion of fibroblasts (line hTERT BJ-5ta). Their growth on the composite surface was 2.4 times more active than on native chitosan. Bulk porous samples of the composition with the weight ratio chitosan-polylactide 50:50 wt. % were synthesized by original method in ammonium bicarbonate presence. Samples were characterized by a porosity of 82.4% and an average pore size of 100 microns. The biodegradability of such material and absence of inflammatory processes were proven in vivo by the blood parameters of experimental animals. Thus, materials with the weight ratio chitosan-polylactide 50:50 wt. % are promising for potential use in regenerative medicine.

## 1. Introduction

Mixtures of polymers are an attractive alternative for the production of new polymeric materials with desired properties, without the need to synthesize completely new materials. Other advantages of obtaining mixed compositions are versatility, simplicity, and low cost of their production. Composites and copolymers of polysaccharides with polyhydroxyalkanoates, such as chitosan and polylactide, are of great practical and theoretical interest for biomedicine [1,2].

Chitosan is a deacetylated form of the natural biopolymer of chitin [3] and consists mainly of 2-amino-2-deoxy-β-d-glucopyranose (d-glucosamine) units. Chitosan is considered the most promising polysaccharide for biomedical applications [4]. The unique structure and properties of this natural cationic polymer make it a valuable starting material for the synthesis of graft copolymers. Chitosan and its derivatives show excellent biocompatibility, biodegradability, absence of allergic reactions, and inflammatory processes [5,6]. The use of chitosan as a component of cartilage scaffolds seems to be a promising approach to enhancing chondrogenesis [7]. Recently, chitosan has been considered as one of the most promising biomaterials for vascular surgery, tissue regeneration and also as a hemostatic agent [8,9]. However, chitosan materials have insufficient mechanical properties, which limits their use in biomedicine. This can be solved by the addition of biodegradable polyhydroxyalkanoates such as polylactide or polycaprolactone, which have good mechanical properties.

Polylactide (PLA) [10] is actively used for biomedical applications as threads, screws, intraosseous and soft tissue implants, as well as vectors for the long-term release of bioactive compounds due to its good mechanical properties, biocompatibility, and biodegradability [11]. Copolymers of polylactide with polyglycolic acid have an increased rate of degradation in the body due to the low crystallinity of the copolymer, which expands the possibilities of its use for biomedical purposes [12]. The absence of functional groups (–NH_2_, OH) that promote cell adhesion and the possibility of developing an inflammatory reaction during the enzymatic decomposition of polylactide in the body limits its use in tissue engineering.

Along with this, the problem of the synthesis of polylactide, which does not require laborious operations to remove the catalyst, and also with high physical and mechanical characteristics of the product, remains urgent [13]. There are two fundamentally different approaches. The first is the use of lactic acid polycondensation; the second is the ring-opening polymerization of lactide [14]. The process of polycondensation of lactic acid can be carried out both in the mass of the monomer and in solution. The disadvantage of this synthesis of polylactide is the low molecular weight of the resulting polymer (M_w_ < 3.5 × 10^4^), or high values of the polydispersity index (M_w_/M_n_ > 4), as well as the need to maintain a high synthesis temperature (160–180 °C), but the advantage of this method is the absence of the need to use catalysts can be distinguished [15]. The second method for the synthesis of polylactide is ring-opening polymerization (ROP polymerization) [16]. The most common catalyst for the ROP polymerization reaction is Sn(Oct)_2_ [17]. Despite the high efficiency of the catalyst, polylactide obtained by this method is contaminated with tin and increases the cytotoxicity of the material, which requires an additional procedure for thorough purification of the polymer from the catalyst [18].

Extensive studies on the polymerization of lactide are carried out in the direction of using metal alkoxides, in which all alkoxy groups are involved in the initiation and further growth of chains [19]. The work [20] discusses the reactivity of alkoxy groups in polymerization reactions. It has been found that metal alkoxides such as Al(O^i^Pr)_3_ and Ti(O-iPr)_4_ are capable of forming various structures that differ in reactivity. The use of titanium alkoxides as catalysts is most justified since they are less toxic than aluminum alkoxides and are more readily available. The authors suggest that the high catalytic activity of titanium alkoxides during the polymerization of lactide is due to the formation of complexes [21]. In [22], the possibility of synthesizing polylactide by using titanium alkoxides as polymerization catalysts was shown; the resulting polylactide was characterized by low molecular weight (M_w_ = 35–40 × 10^3^) and a polydispersity index (M_w_/M_n_ = 1.7–2.2). In [23], the polymerization of lactide on chlorine-substituted titanium isopropoxide is considered. The molecular weight of polylactide was (85–90 × 10^3^) and the polydispersity index (1.3–1.5), but the synthesis was characterized by a low reaction yield (20–30%). ROP-polymerization by titanium-based catalysts with dpp-bian ligands with a high yield of the target product was studied in [24]. Polylactide had a polydispersity index (M_w_/M_n_ = 1.1–2.5) and a molecular weight (M_w_ = 40 × 10^3^). Thus, the problem of the synthesis of polylactide with a high molecular weight, a low polydispersity index, and a high yield of the target product remains urgent [25].

As noted above, it can be expected that hybrid materials based on natural and synthetic polymers will synergistically combine the useful functionality of their constituent polymers and neutralize disadvantages. Studies of the morphology of mixtures of chitosan and polyhydroxyalkanoates have confirmed that they are systems with phase separation, therefore there is a problem associated with their compatibility, as well as the unsuitability of polysaccharides for processing in the molten state, which prevents the development and production of many promising polymer systems based on polysaccharides [26,27]. Recently, several polymeric materials based on chitosan and polylactide in the form of films, porous scaffolds, micro- and nanoparticles have been obtained and studied from the point of view of their use for biomedicine [28,29,30].

One of the methods for combining such polymers is reactive mixing of polymers using high shear stresses. Thus, when chitosan is combined with polylactide using a twin-screw extruder, a graft copolymer is formed [31,32]. The yield of graft copolymers, which contribute to the compatibility of the components in this melt processing, is negligible. Grafting occurs on the very surface of the polysaccharide particles. The size of chitosan particles practically does not change compared to the initial ones [33]. The material obtained by this method has good biocompatibility and biodegradability; however, since polylactide is grafted only on the surface of particles, toxic solvents such as chloroform and acetone must be used to combine these polymers. It is possible to obtain a thermoplastic mixture based on polylactide and chitosan with the addition of glycerol and water. The use of plasticizers leads to the deterioration of cell adhesion and hydrolysis of the polyhydroxyalkanoate matrix, and the elevated temperatures (130–150 °C) required for mixing polymers lead to partial destruction of chitosan [34]. Another option for combining chitosan and polylactides is the electrospinning method. As a result, a biocompatible and biodegradable nonwoven material is obtained in the form of films, the strength of which is sufficiently low (σ < 5 MPa) for use in tissue engineering [35]. In [36], synthesis was carried out to obtain a graft copolymer of chitosan-polylactide. The reaction was carried out in a solution of dimethyl sulfoxide, with the formation of a suspension of chitosan, which provided copolymerization only on the surface of the particles, as a result of which the grafting efficiency was about 40%. Thus, the growing needs of medicine and biotechnology require the development of approaches to obtaining materials based on chitosan and polylactide with high physical and mechanical characteristics.

The aim of the work was to create mixed compositions based on chitosan and polylactide, to study their mechanical, structural, and biological properties. This study delivers some insights into the synthesis of such materials and their characterization, setting a benchmark for further developments of material for regenerative medicine based on chitosan–polylactide blends.

## 2. Materials and Methods

### 2.1. Materials

For materials obtaining in this research, we used chitosan with viscosity average molecular weight (M_η_) 200 × 10^3^ and degree of deacetylation 82% (source–crab, LLC Bioprogress, Moscow, Russia); d,l-lactide (ACROS Organics, Geel, Belgium); titanium tetraisopropoxide (“ACROS Organics”, Geel, Belgium, the content of the main substance is 98%, without additional purification); acetic acid (99%, Khimreaktiv, Moscow, Russia) and dioxan (chemically pure, Khimreaktiv, Moscow, Russia). The viscosity average molecular weight of chitosan was determined by viscosimetry. Measurements were carried out on an Ubbelohde viscometer at 21 °C in a solution of acetic acid with a concentration of 0.33 base-mol/L in distilled water containing 0.3 mol/L NaCl. The viscosity average molecular weight was calculated using the Mark–Kuhn–Houwink equation $\left[\eta \right]=k\cdot M_{\eta }^{\alpha }$, where k = 3.41 × 10^−5^ and α = 1.02 [37]. The degree of deacetylation of chitosan was determined by potentiometric titration of its solutions in a 0.1 N HCl solution with NaOH solution (0.1 N) using an pH meter (FiveEasy Plus pH meter FP20-Std-Kit, Mettler Toledo, Columbus, OH, USA).

### 2.2. The Technique of Obtaining Polylactide by Ring-Opening Polymerization

Polylactide (PLA) was obtained by ring-opening polymerization; titanium isopropoxide was used as a catalyst. The ring-opening polymerization of lactide was carried out at different molar ratios of the components. For this, a mixture of lactide and titanium isopropoxide was heated at 130 °C for 24 h. During the process, samples were taken 8, 16, 20, and 24 h after the start of the synthesis. The polylactide was precipitated with ethanol and dried to constant weight under vacuum.

### 2.3. X-ray Fluorescence Analysis

Elemental analysis of PLA was carried out on a scanning electron microscope (JSM-IT300LV, JEOL, Akishima, Tokyo, Japan) with an X-ray fluorescence attachment with an energy of 20 kV.

### 2.4. IR Spectroscopy

The IR spectrum of PLA was obtained using tablets based on potassium bromide mixed with samples on a FTIR spectrophotometer (Shimadzu IR Prestige-21, Kyoto, Japan).

### 2.5. Determination of Molecular Weight Characteristics

The specification of the molecular weight characteristics of PLA was carried out on a Shimadzu gel permeation chromatograph (Shimadzu Corporation, Kyoto, Japan) with columns filled with polystyrene gel with pore size 1 × 10^5^ and 1 × 10^4^ Å. A differential refractometer was used as a detector. Chromatograms were processed using LCsolution software (version 1.25, Shimadzu Corporation, Kyoto, Japan). Narrowly dispersed polystyrene standards were used for calibration.

### 2.6. Determination of the Optical Angle of Rotation

The angle of optical rotation and the specific optical rotation of polymer solutions in chloroform were determined on an ATAGO AP-300 (Atago, Tokyo, Japan) automatic polarimeter at a wavelength of the D line of the sodium spectrum (λ = 589.3 nm) at a temperature of 20–25 °C, and a tube length of L = 99.96 mm. The number of D-units (D%) in polylactide was calculated by the formula:

(1) $$D\%=\frac{\left({\left[\alpha \right]}_{D}^{25}\left(\mathrm{PLLA}\right)-{\left[\alpha \right]}_{D}^{25}\left(\mathrm{PLA}\right)\right)}{2{\left[\alpha \right]}_{D}^{25}\mathrm{PLLA}}\times 100\%,$$

where ${\left[\alpha \right]}_{D}^{25}\mathrm{PLA}\;\mathrm{and}\;{\left[\alpha \right]}_{D}^{25}\mathrm{PLLA}$ are the specific optical rotation of polylactide and poly-l-lactide in the sample, respectively.

The measurements for the samples of each of the syntheses were repeated three times, and then the arithmetical mean value of α were determined.

The average value of α was used to determine the specific rotation of the obtained polymers. All measurements were carried out in chloroform solution for comparability of experimental data.

### 2.7. Obtaining Polymer Films Based on a Mixed Composition of Chitosan and Polylactide

Mixed compositions of chitosan and PLA were prepared using a 3% solution of chitosan in 1.5% lactic acid and PLA solutions in dioxane of various concentrations. The PLA solution was poured into the chitosan solution dropwise with continuous stirring, which contributed to the formation of a homogeneous system. Polymer films with different ratios of chitosan and PLA were obtained on a polyethylene terephtalate substrate of a Xiamen TMAX-TMH (Xiamen, China) film-filling machine. For further research, chitosan in films was converted from the salt to the basic form.

### 2.8. X-ray Phase Analysis

X-ray phase analysis of the samples was performed on a Bruker D8 Discover X-ray diffractometer (Bruker, Billerica, MA, USA) using CuKα radiation. Diffraction patterns were recorded for an angular range of 10–60° in terms of diffraction angle 2θ in symmetric geometry with a 0.6 mm slit on the primary beam and a LynxEye linear position-sensitive detector.

### 2.9. Mechanical Properties

Mechanical characteristics (tensile strength and elongation) of the material were determined on a Zwick/Roell Z005 (Zwick/Roell GmbH, Ulm, Germany) universal testing machine in accordance with ASTM D 882. The tests were carried out at a tensile rate of 10 mm/min on specimens 60 ± 5 μm thick in the form of rectangles 15 mm wide. The clamping length was set to 30 mm. For the films of each composition, at least ten samples were measured.

### 2.10. Dynamic Mechanical Analysis (DMA)

The temperature dependences of the elastic modulus of the films were studied by the DMA method on a DMA 242C/1/F setup (Netzsch, Selb, Germany). The measurements were carried out at a loading frequency n = 1, 2, 5, and 10 Hz, an amplitude of 40 μm, and a heating rate of 5 °C/min. Before testing, the films were dried in a vacuum oven for 24 h.

### 2.11. Preparation of Porous Samples Based on Chitosan and Polylactide

Porous samples were obtained based on a solution of chitosan and polylactide, using ammonium bicarbonate as a pore-forming agent. It is essential that along with the formation of a porous structure, chitosan is converted from the salt form to the basic one. The obtained samples were washed with alcohol and dried in a vacuum oven to constant weight.

### 2.12. Mercury Intrusion Porosimetry

The porosity, size, and pore distribution of the samples were determined using a Pascal 140 and 440 mercury intrusion porosimeter (ThermoFisher Scientific, Rodano, Italy). The use of a unique ultra-macroporous dilatometer makes it possible to analyze the pore sizes from 3.6 nm to 1200 μm. For measurements, the samples were cut into small pieces (100–200 mg).

### 2.13. Investigation of the Biocompatibility of Samples Based on Chitosan and Polylactide

To evaluate the biocompatibility of films adhesion, cytotoxicity and growth of cells on the surface of the film were studied during the cultivation of human fibroblasts of the hTERT BJ-5ta cell line. Films of material, after sterilization by autoclaving at 110 °C, were placed in the wells of a cell culture plate and filled with 500 μL of DMEM medium. The cells were seeded onto the surface of the film with a density of 1.6 × 10^5^/cm^2^ and cultured for 24 h. Cell visualization and assessment of their viability were assessed by fluorescence microscopy. Fibroblasts were stained by 2 × 10^−4^ wt. % solution of acridine orange in phosphate buffer. This dye, by intercalation or electrostatic attraction, selectively interacts with DNA and RNA located in the nucleus and mitochondria of the cell, respectively. This allows assessing the general state of cells—activity, proliferation, and apoptosis. Microsampling of the films was carried out on an Olympus IX71 inverted microscope (Japan/Germany) using a filter (emission wavelength 510–555 nm, excitation wavelength 460–495 nm), which allows visualizing the green color of the nucleus of living cells.

The study of biodegradability of porous samples was carried out on white nonlinear female rats of three months of age weighing 150–200 g. The obtained samples were implanted in experimental animals in the interscapular region of the back. All procedures on laboratory animals were carried out in accordance with the requirements of the European Convention for the Protection of Vertebrate Animals used for Experimental or Other Scientific Purposes (Strasbourg, 18 March 1986); of the Rules of laboratory practice in the Russian Federation (order of the Ministry of Health of the Russian Federation No. 267 of 19 June 2003). The biodegradability of the material was assessed by the change in the mass of the implanted samples.

Experimental animals were divided into four groups of 7 animals each: Group 1—intact animals (relative norm); Group 2—control (the animals underwent an incision without introducing the sample); Group 3—the animals were injected with porous samples based on pure chitosan; Group 4—the animals were injected with porous samples based on chitosan-polylactide. A skin incision was made in the interscapular region of the pre-anesthetized rats of Groups 2, 3, and 4.

To assess the effect of the implanted samples on the functional state of animals, the possible development of inflammatory processes, and an allergic reaction in them, 7 and 21 days after the introduction of the film, blood was taken from the rats for analysis. In the blood, the number of leukocytes, erythrocytes, and hemoglobin was determined using the AbacusJunior 30 hematological analyzer (Diatron, Austria). The percentage of lymphocytes, neutrophils, eosinophils, and monocytes was determined on blood smears stained according to the conventional Romanovsky-Giemsa method. The results were statistically processed using the BioStat program. To determine statistically significant differences in blood tests between intact, control and experimental groups of animals, multiple comparison methods were used applying the Student’s t test with the introduction of the Bonferroni correction, as well as with its nonparametric analog, which does not require the assumption of normal distribution—the Kruskal–Wallis test [38]. Animal experiments were performed three times. The type of uncertainty used was confidence interval. The state of local inflammation was assessed by the absence or presence of a fibrous capsule around the sample. Bioresorbability was assessed by the decrease in the weight of the injected sample 1 and 3 weeks after administration.

## 3. Results and Discussions

### 3.1. Study of the Ring-Opening Polymerization Reaction of Lactide

**Figure 1:**
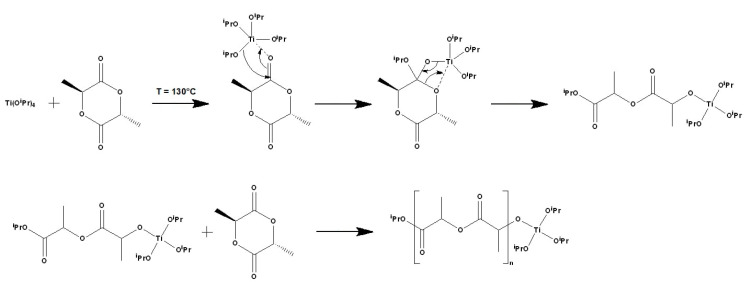
Proposed scheme for the polymerization of lactide Temperature of reaction—130 °C. ^i^Pr = isopropyl.

As noted above, the production of polylactide with a high molecular weight and under conditions excluding the use of toxic catalysts is relevant for use in biomedicine. In this regard, a technique was developed that ensures the production of polylactide using titanium isopropoxide as a catalyst [39]. The proposed reaction scheme is shown in Figure 1.

The process was carried out at different molar ratios of lactide (LA) and titanium isopropoxide (TTIP). The resulting products were precipitated with ethanol and dried in a vacuum oven to constant weight. The molecular weight characteristics of PLA are shown in Table 1.

As can be seen from Table 1, the conversion rate slightly depends on the molar ratio of the components and, at the same time, significantly affects the molecular weight and the polydispersity index of the product. The reaction yield for all variants of the mixture varies from 70% to 85%, however, with a molar ratio of TTIP to LA of 1:3, respectively, the best results in terms of molecular weight and polydispersity index are observed. Therefore, the dependence of the conversion depth in time and the further synthesis of polylactide for research was carried out according to this option. The results are shown in Table 2.

To increase the molecular weight, for the reaction mixture with a molar ratio of TTIP to LA 1:3, the reaction time was extended to 36 h. However, the molecular weight of the resulting polylactide remained practically at the same level as after 24 h and was 81 × 10^3^. Therefore, maintaining the reaction mixture for 24 h can be considered the most effective.

**Figure 2:**
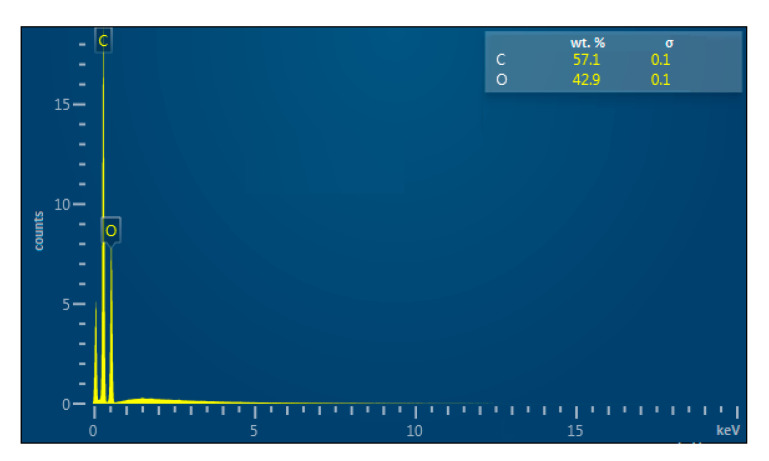
Elemental composition of polylactide.

It is known that titanium alkoxides can form complex polycondensation structures, which can be assumed to be included in the composition of the polymer. Therefore, in order to study the structure of the polymer and the effect of the catalyst on the composition, the obtained polymer was examined for elemental composition using an X-ray fluorescence attachment of an electron microscope (Figure 2).

As can be seen from Figure 2, the elemental composition of the obtained polylactide, in terms of carbon and oxygen content, agrees with the results for the control sample of pure polylactide. The titanium content in the sample is less than 0.1%, which indicates that titanium isopropoxide acts as a catalyst and is practically not included in the resulting polymer.

It is known that polylactide can exist both in the form of individual optical isomers (PLLA or PDLA), which are polymers with a high degree of crystallinity and in the form of a racemate (PLA). As noted in [32], the biological and mechanical properties of polylactide depend on the stereo directionality, which can be determined from the angle of rotation. The results of calculation of the content of L-units are presented in Table 3.

The content of L-units in polylactide, determined by the polarimetry method, coincides with that calculated from the composition of the monomer mixture of d,l-lactide, and proves that the reaction product is poly-d,l-lactide.

**Figure 3:**
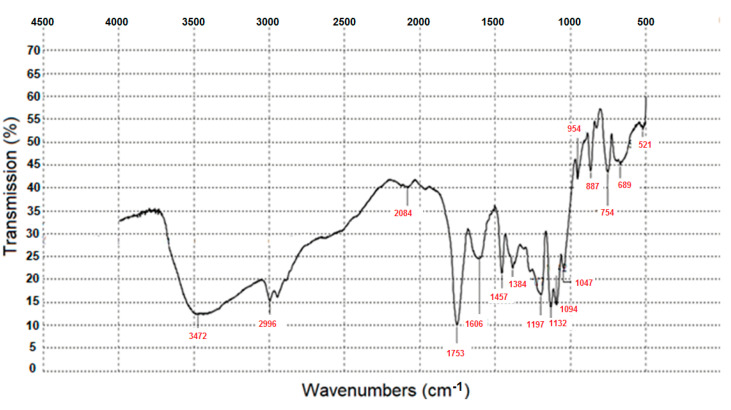
IR spectrum of the obtained polylactide.

The data of IR spectroscopy (Figure 3) is the evidence that polylactide was obtained as a result of the synthesis.

In the IR spectrum of the synthesized PLA, the characteristic bands are clearly visible: stretching vibrations –CH in the CH_3_ group, a band at 2996 cm^−1^ (symmetric vibrations); stretching vibrations of the carbonyl group C=O (band 1753 cm^−1^) and stretching vibrations of oxygen (in the C–O–C group), with several bands in the range from 1047 cm^−1^ to 1197 cm^−1^.

### 3.2. Obtaining Polymer Films Based on Mixed Compositions of Chitosan-Polylactide

Polymer films with different ratios of chitosan and polylactide were obtained at the following mass ratios chitosan to PLA: (3:1), (2:1), (1:1). The introduction of a larger amount of polylactide led to the stratification of the system and did not allow the formation of samples for further research.

Since the obtained films are intended for use in biomedicine, chitosan was converted from a salt form to a basic one. For this, the films were treated with an aqueous solution of NaOH for 2 h, after which they were washed with distilled water, bringing to a pH value of 7.

### 3.3. Mechanical Properties of Films Based on Chitosan and Polylactide

The materials used in tissue engineering must have mechanical properties comparable to the tissue being replaced. The measurement of the mechanical properties of samples in the form of films, based on a mixed composition of chitosan-polylactide (Table 4) was carried out.

**Figure 4:**
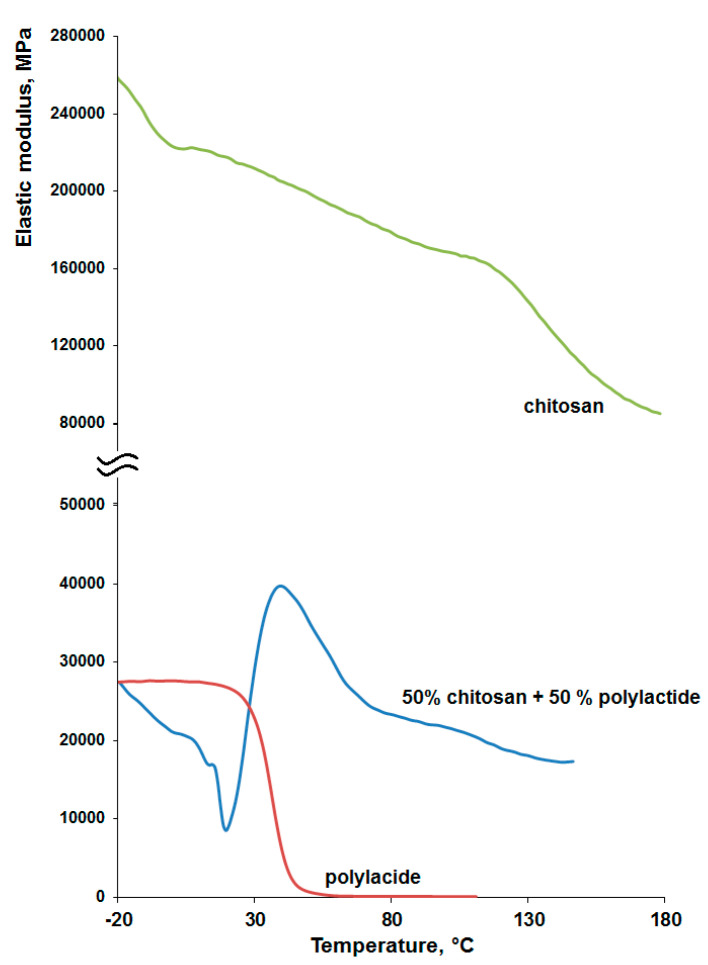
Dynamic mechanical analysis of samples.

It can be seen from Table 4 that the mechanical characteristics significantly depend on the ratio of the components, reaching the maximum value for the composition of 50% chitosan with 50% PLA, by weight. As noted above, the introduction of polylactide in an amount greater than 50% led to phase separation of the system and the impossibility of forming homogeneous films. Since material has to replace damaged tissue, samples with composition “50% chitosan + 50% PLA” were chosen for further research, because they meet the requirements for tissue engineering and have the strength indicators that are closest to those of human skin (tensile strength—27.2 ± 9.3 MPa and deformation 25.4 ± 5.1%) [40,41]. The high strength characteristics of the films of the specified composition are confirmed by the DMA data (Figure 4).

Figure 4 shows that chitosan has a large modulus of elasticity. This polymer is rigid and cannot withstand mechanical loads during bending. So, chitosan cannot fully provide the necessary tensile strength and elasticity of wound-healing materials, which is inherent in all polysaccharides. The polylactide modulus of elasticity in the temperature range from 30 °C to 42 °C drops sharply, which can lead to instability of the properties of the material in the body. On the contrary, the obtained composition is characterized by an increase in the elastic modulus in the specified temperature range, and a further decrease in the elastic modulus is insignificant. In our opinion, the observed effect can be explained as follows. The mobility of polylactide segments in the chitosan-PLA composite increases when the sample is heated above the PLA glass transition temperature under deformation conditions under dynamic load. This leads to a weakening of the Van-der-Waals interaction between PLA chains. At the same time, in this case, the interaction of functional groups of chitosan (amino and hydroxyl) and polylactide(carbonyl) chains increase with the formation of hydrogen bonds throughout the sample volume. Thus, due to them, a three-dimensional structure is formed in the sample, which leads to an increase in its elastic modulus in the temperature range of 20–60 °C. These mechanical properties make it promising for use in tissue engineering [42].

**Figure 5:**
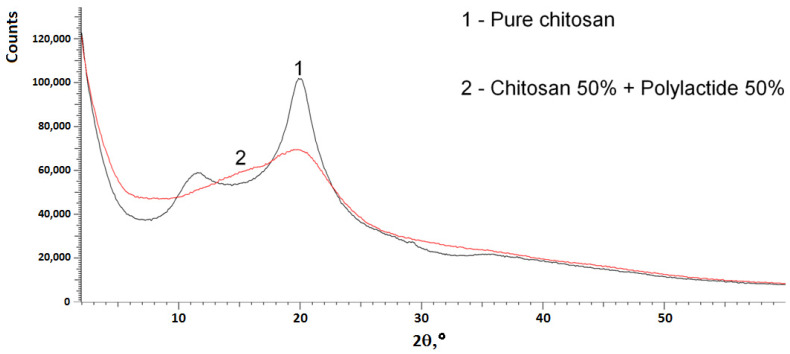
X-ray phase analysis of samples.

The high strength characteristics of the chitosan-polylactide composition are apparently due to a change in the structure of chitosan in a mixture with polylactide, which is confirmed by X-ray phase analysis data (Figure 5).

It can be seen from Figure 5 that in the samples of films of pure chitosan, characteristic intense peaks are observed in the region of 10° and 20° in 2θ corresponding to the crystallographic planes (002) and (101), respectively, indicating the presence of crystalline structures. A detailed analysis of polymorphic forms of chitosan is given in [43]. The crystalline peak centered at around 10° is attributed to the hydrated crystalline structure of chitosan while the crystalline peak at 20° is an indication of the relatively regular crystal lattice of chitosan [43,44]. It is known that chitosan always contains bound water even when extremely dried. The incorporation of bound water molecules into the crystal lattice, commonly termed hydrated crystals, generally gives rise to a more dominated polymorph which can normally be detected by a broad peak in the corresponding XRD patterns. The disappearance of the peak at 10° and the appearance of an amorphous halo in the same range of angles are observed in the diffractogram of a sample based on chitosan and polylactide. Apparently, there is an opening of hydrogen bonds between hydrated chitosan macromolecules first and then a spatial network formation due to hydrogen bonds between the OH-groups of chitosan and carbonyl groups of polylactide with the participation of bound water, which facilitates this interaction.

**Figure 6:**
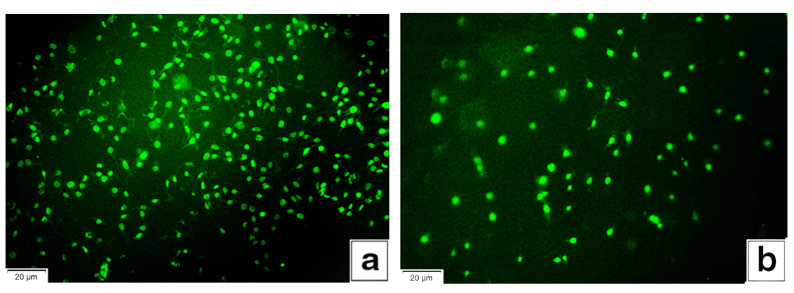
Micrographs of films, 24 h after colonization with fibroblasts. (**a**) A film based on a mixed composition of the composition “50% chitosan + 50%PLA”; (**b**) a film based on pure chitosan.

In vitro studies of films were carried out on the adhesion and proliferation of fibroblast cells as precursors of connective tissue for the composition of chitosan-polylactide, pure chitosan, and polylactide. Figure 6a,b shows photographs of films after 24 h of cell incubation. As can be seen from the figures, the film based on the composition of chitosan and polylactide is characterized by a uniform distribution of fibroblasts on the surface, active growth and cell division, which indicate a high degree of cell adhesion and biocompatibility. The properties obtained are significantly higher than those of films made of pure chitosan. This indicated a higher degree of biocompatibility and cell adhesion in comparison with the pure chitosan film. To compare the growth of cells on the surface of the films, we used the ImageJ software (National Institutes of Health, Bethesda, MD, USA). The growth of fibroblast cells on the surface of the modified chitosan film was 2.4 times more active than on pure chitosan.

Fibroblast adhesion was not observed on polylactide films under experimental conditions. Thus, the totality of the results for the chitosan-polylactide composition makes it promising for the manufacture of wound-healing film materials with increased regeneration of damaged tissue.

The set of results on mechanical and biological properties suggests that it is promising to obtain three-dimensional porous structures based on a chitosan-polylactide composition as a polymer component of scaffolds.

**Figure 7:**
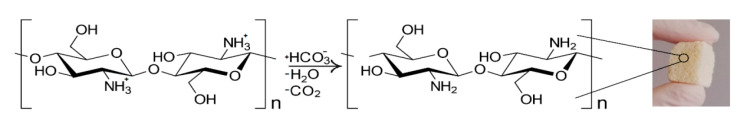
Scheme of the reaction of the interaction of chitosan with ammonium bicarbonate.

A process for the formation of a three-dimensional structure has been developed using ammonium bicarbonate as a pore-forming agent. The salt form of chitosan, with similarly charged protonated amino groups of chitosan, complicates the formation of a three-dimensional structure. The originality of the method lies in the fact that simultaneously with the formation of a porous structure, the transition of chitosan to the basic form occurs. The mechanism of the process is based on the exchange reaction between the anions of the acetate group of chitosan and bicarbonate with the release of carbon dioxide as a result of the decomposition of carbonic acid, simultaneously accompanied by the formation of a three-dimensional structure due to the intermolecular and intramolecular hydrogen bonds in the basic form of chitosan (Figure 7).

**Figure 8:**
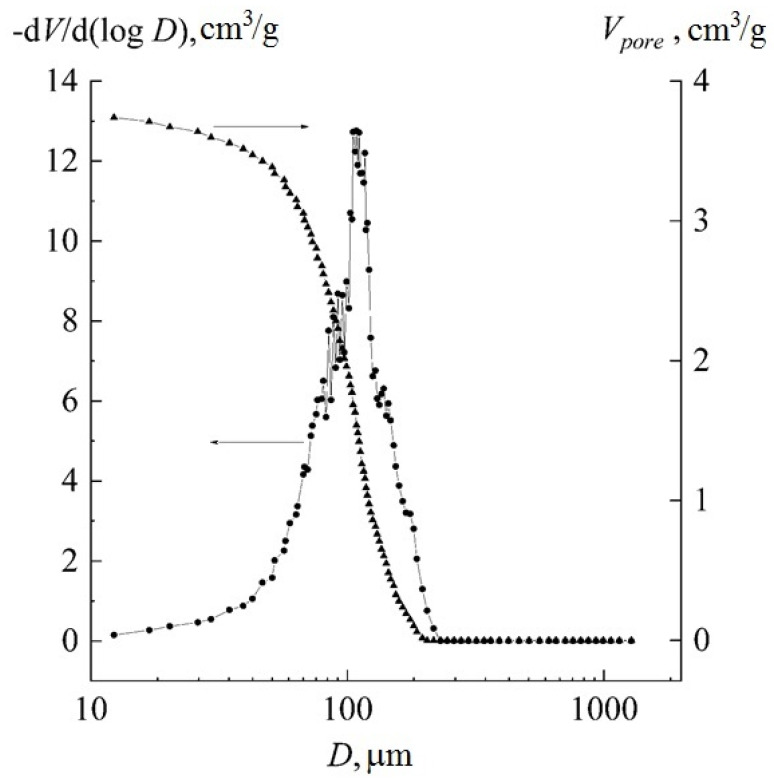
The pore size distribution of chitosan–polylactide blend.

As was shown in [33], the porosity and pore size of the polymer component of the scaffold are important in the preparation of bone-replacing materials. The porosity and pore size were determined by mercury intrusion porosimetry (Figure 8). It can be seen from the figures that the obtained porous samples are characterized by high porosity and have an average pore diameter of about 100 μm, which facilitate cell proliferation and vascularization.

The porous materials were tested for biocompatibility and biodegradability in in vivo experiments. Cylinder-shaped implants were inserted into an incision under the skin in the interscapular region of laboratory animals. The functional state of the animals was determined by blood parameters.

A week after implantation, the same type of changes in blood parameters was observed in the control and experimental groups. The number of leukocytes, lymphocytes, neutrophils was practically the same in all experimental groups (Table 5). The number of eosinophils in the experimental groups did not exceed the values in intact and control animals. These indicators testified that the immune system of the experimental animals did not react to the material of the implant. The response occurred with a high degree of probability of surgery (skin incision). An increased number of monocytes, one of the functions of which is preparation for wound healing, confirm the organism’s response to surgery. Table 5 shows that a decrease in the number of lymphocytes, and an increase in the number of neutrophils a week after the introduction of the implant are not observed. This is the criterion that the rats were not under stress.

The results of blood counts of animals three weeks after implantation are presented in Table 6. As in the previous time intervals, no allergic response to the implant material was noted, and the animals were not under stress.

After removal of the implants, no fibrous capsule formation was observed, indicating no rejection but biocompatibility. Weighing the samples before and after implantation revealed a decrease in their masses, which indicated their biodegradability (Table 7).

## 4. Conclusions

A biocompatible and biodegradable composition based on chitosan and poly-d,l-lactide has been obtained. The method for obtaining poly-d,l-lactide with a molecular weight of up to 80 × 103 and a high yield of the target product has been worked out. The composition of 50% of chitosan and 50% of PLA is characterized by high mechanical properties, with a breaking stress of ~70 MPa, with deformation of 10%, and an elastic modulus of 40 GPa. Three-dimensional porous samples obtained by the original technique have sufficient porosity and pore diameter to be used as a polymer component of scaffolds in tissue engineering. The results of the study in experiments in vivo and in vitro showed the absence of cytotoxicity and rejection of the samples under study, and the increased cell adhesion and cell growth on film samples indicate the prospects of using the composition in tissue engineering for two-dimensional and three-dimensional materials for wound surface coatings and treatment of injuries of various etiologies as tissue-containing matrices.

## Figures and Tables

**Table 1:** Molecular weight characteristics and yield of the reaction product.

Composition	LA:TTIP (3:1)	LA:TTIP (5:1)	LA:TTIP (10:1)	LA:TTIP (20:1)
M_w_ × 10^−3^	80.1 ± 7.5	60.7 ± 8.4	35.3 ± 3.7	5.2 ± 0.5
M_n_ × 10^−3^	64.5 ± 5.8	38.7 ± 5.2	20.7 ± 2.5	2.6 ± 0.2
M_w_/M_n_	1.24 ± 0.21	1.55 ± 0.23	1.75 ± 0.38	1.91 ± 0.31
Reaction yield (%)	79.2 ± 5.4	77.8 ± 4.7	84 ± 2.3	68.3 ± 5.8

**Table 2:** Conversion depth and molecular weight characteristics during PLA synthesis.

Time, Hours	8	16	20	24
M_w_ × 10^−3^	4.5 ± 0.6	50.4 ± 6.2	63.0 ± 5.8	8.1 ± 7.5
M_n_ × 10^−3^	2.7 ± 0.3	32.3 ± 3.4	42.6 ± 3.5	64.5 ± 5.8
M_w_/M_n_	1.7 ± 0.3	1.6 ± 0.4	1.5 ± 0.3	1.2 ± 0.2
Conversion depth, %	4.9 ± 0.8	14.8 ± 1.4	39.6 ± 3.1	79.2 ± 5.7

**Table 3:** Polylactide polarimetry results.

l-Lactide Content in Monomer Mixture, % Mass.	$\mathrm{Rotation}\;\mathrm{Angle},\;{\left[\alpha \right]}_{D}^{25}$	Content of L-Units in Polylactide, %
50 (d,l-lactide)	−48 ± 3	50 ± 3
100 (l-lactide)	−153 ± 1	99 ± 1

**Table 4:** Physical and mechanical characteristics of samples.

Composition of the Film	Tensile Strength, σ (MPa)	Deformation, ε (%)
100% chitosan	15.5 ± 1.3	2.5 ± 0.3
100% PLA	32.9 ± 1.8	42.7 ± 8.6
75% chitosan + 25% PLA	42.2 ± 5.1	6.9 ± 0.7
67% chitosan + 33% PLA	58.4 ± 6.0	7.9 ± 1.1
50% chitosan + 50% PLA	72.1 ± 8.2	10.3 ± 1.9

**Table 5:** Blood parameters of rats one week after implantation.

Indicators	Intact	Control	Group 1 Chitosan 100%	Group 2 Chitosan 50% + PLA 50%
Leukocytes (×10^9^ cells/L)	12.2 ± 1.0	10.9 ± 1.2	9.1 ± 0.5 *	10.1 ± 1.0
Lymphocytes, %	48.8 ± 2.1	54.8 ± 6.6	53.9 ± 3.2	60.9 ± 3.7
Monocytes, %	9.9 ± 1.1	16.2 ± 1.6 *	14.0 ± 0.9 *	10.9 ± 0.7 **
Neutrophils, %	37.8 ± 2.0	26.6 ± 2.1 *	28.9 ± 1.7 *	27.1 ± 2.8 *
Eosinophils, %	3.6 ± 1.1	2.5 ± 1.0	2.2 ± 0.9	1.0 ± 0.4 *
Erythrocytes (×10^12^ cells/L)	4.7 ± 0.7	4.9 ± 0.3	4.9 ± 0.3	4.1 ± 0.2 **
Hemoglobin (g/L)	103.6 ± 12.6	103.9 ± 4.0	104.6 ± −6.3	88.1 ± 5.5 **

Note: *—statistically significant differences (*p* ≤ 0.05) relative to the intact group, **—statistically significant differences (*p* ≤ 0.05) relative to the control group.

**Table 6:** Blood parameters of the same rats three weeks after implantation.

Indicators	Intact	Control	Group 1 Chitosan 100%	Group 2 Chitosan 50% + PLA 50%
Leukocytes (×10^9^ cells/L)	11.7 ± 1.1	8.8 ± 2.5 *	9.5 ± 0.1 *	11.6 ± 1.0
Lymphocytes, %	47.6 ± 2.3	62.4 ± 3.6 *	52.1 ± 3.1	63.2 ± 2.1 *
Monocytes, %	10.1 ± 0.9	10.8 ± 1.3	12.5 ± 0.4 *	9.1 ± 0.1
Neutrophils, %	38.5 ± 1.2	26.2 ± 3.7 *	34.05 ± 1.7 **	25.45 ± 1.1 *
Eosinophils, %	3.5 ± 1.1	0.6 ± 0.4 *	1.25 ± 0.9	2.25 ± 0.8 **
Erythrocytes (×10^12^ cells/L)	4.5 ± 0.9	5.8 ± 0.4	5.72 ± 0.3	6.26 ± 0.3 *
Hemoglobin (g/L)	104.6 ± 11.8	125.6 ± 11.9	130.8 ± 3.8 *	137.6 ± 4.8 *

Note: *—statistically significant differences (*p* ≤ 0.05) relative to the intact group, **—statistically significant differences (*p* ≤ 0.05) relative to the control group

**Table 7:** Change in sample weight during implantation.

Composition	Mass of the Sample, g			
	Initial	In 1 Week	In 2 Weeks	In 3 Weeks
Chitosan	1.15 ± 0.61	0.77 ± 0.06	0.68 ± 0.06	0.25 ± 0.03
50% Chitosan + 50% PLA	1.18 ± 0.62	0.77 ± 0.07	0.68 ± 0.07	0.28 ± 0.03

## Data Availability

Data sharing not applicable.
